# Validation of Reference Genes for RT-qPCR Normalization in *Saccharum* Genotypes During Smut Infection

**DOI:** 10.3390/ijms27219393

**Published:** 2026-10-22

**Authors:** Laísa M. Rocha, Danyel F. Contiliani, Vanessa Thomé, Claudia B. Monteiro-Vitorello, Silvana Creste

**Affiliations:** 1 Graduate Program in Genetics, Ribeirão Preto Medical School, University of São Paulo, Ribeirão Preto, SP 14001-970 , Brazil; laisarocha@usp.br; 2 Sugarcane Center, Agronomic Institute (IAC), Ribeirão Preto, SP 14032-800, Brazil; danyelfc1@gmail.com (D.F.C.); vanessa.thome@alumni.usp.br (V.T.); 3 Department of Genetics, Luiz de Queiroz College of Agriculture, University of São Paulo, Piracicaba, SP 13418-900, Brazil; cbmontei@usp.br

**Keywords:** quantitative real-time PCR, differential expression, normalization, reference gene, fungus, *Sporisorium scitamineum*, sugarcane, energy cane

## Abstract

Sugarcane and energy cane (*Saccharum* spp. hybrids) yields are significantly threatened by smut disease, caused by the fungus *Sporisorium scitamineum*. Gene expression studies are essential for understanding molecular responses in this pathosystem; however, reference gene stability may vary among genotypes, plant organs, and across infection stages, highlighting the need for systematic validation under the specific conditions of each experiment. In this study, eight candidate genes were assessed in the buds of sugarcane IACSP02-1064 (intermediate resistance) and energy cane Vertix 10 (susceptible) during the course of the infection (12, 24, 48, and 120 hpi). Expression stability was assessed using geNorm, NormFinder, and BestKeeper algorithms. For IACSP02-1064, *ACAD *(*Acyl-CoA dehydrogenase family member 10*) and *GAPDH* (*Glyceraldehyde-3-phosphate dehydrogenase*) were the most stable genes, whereas *GAPDH*/*ACAD* and *ACAD*/*UBQ2* (*Ubiquitin 2*) were identified as optimal pairs. For Vertix 10, *GAPDH*, *UBQ2*, and *ACAD* showed the highest stability, while *GAPDH*/*UBQ2*, *ACAD*/*GAPDH*, and *ACAD*/*SARMp1* were recommended for normalization. The selected reference genes were further evaluated by analyzing *ScSPL19* (*SQUAMOSA promoter-binding protein-like 19* from *Saccharum* spp.) expression, a member of the SQUAMOSA PROMOTER-BINDING PROTEIN-LIKE (SPL) transcription factor family, whose members have previously been reported to respond to smut infection, using the most and least stable reference gene pairs. This analysis revealed differential temporal regulation of *ScSPL19* when normalized with the most stable reference gene pair (*GAPDH*/*ACAD*). These results support the suitability of this reference gene combination for accurate relative gene expression analysis under the evaluated conditions.

## 1. Introduction

Sugarcane (*Saccharum* spp. hybrid), a member of Poaceae family, is a crop of major economic and nutritional relevance. Cultivated on more than 27 million hectares worldwide, it accounts for approximately 80% of global table sugar production and 40% of biofuel production [1,2]. As a highly efficient C4 plant, sugarcane exhibits elevated photosynthetic capacity and remarkable ability to accumulate biomass and sucrose [3]. In addition, energy cane, a high-fiber sugarcane hybrid, has emerged as a promising feedstock due to its superior biomass yield and suitability for second-generation bioenergy production [4].

Despite its importance, sugarcane productivity is frequently constrained by environmental stressors. These include abiotic factors, such as drought and temperature fluctuations, and biotic agents, including pathogens and pests that compromise crop performance [5,6]. Among the biotic stresses, smut disease, caused by the biotrophic fungus *Sporisorium scitamineum,* remains a critical threat [7]. The pathogen typically infects the bud tissues or apical meristem, inducing metabolic reprogramming that interferes with natural growth and culminates in the emergence of characteristic whip-like structures containing teliospores [8,9]. This disease causes severe industrial losses, with yield reductions ranging from 39% to 56% in plant cane and up to 73% in subsequent ratoon cycles in susceptible cultivars [10].

Gene expression studies have emerged as a valuable approach to elucidate the molecular mechanisms underlying plant responses to this disease. Reverse transcription-quantitative polymerase chain reaction (RT-qPCR) has become an indispensable and highly sensitive tool for quantifying these transcriptional shifts, enabling a deeper understanding of complex plant–pathogen interactions and of how divergent molecular responses among cultivars result in distinct biochemical defense profiles [11,12]. To ensure accurate quantification, however, relative RT-qPCR analyses depend on reliable normalization using stably expressed reference genes.

Previous studies have evaluated candidate reference genes across various sugarcane genotypes and stresses, but the resulting stability rankings are often inconsistent [13,14,15,16,17,18]. These inconsistencies reflect the influence of host genotypes, fungal isolates, and experimental conditions on reference gene stability, emphasizing the need for systematic validation in each study rather than relying on previous reports [14,15]. Furthermore, because the sugarcane–*S. scitamineum* interaction is associated with extensive transcriptional reprogramming, accurate normalization is essential for reliable gene expression analyses.

However, few studies have compared the transcriptional stability of reference genes between sugarcane genotypes with contrasting levels of smut resistance [14,15], and, to our knowledge, none have evaluated this in the sugarcane cultivar IACSP02-1064 (smut-intermediate resistant) and the energy cane genotype Vertix 10 (smut-susceptible). Since comparative gene expression analyses between resistant and susceptible genotypes are important for elucidating the molecular basis of smut resistance, careful validation of reference genes is required to ensure accurate normalization within and across genotypes, thereby enabling reliable comparisons of transcriptional responses to infection.

In this study, we evaluated the transcriptional stability of eight candidate reference genes, previously reported in *Saccharum* spp., using the widely adopted algorithms BestKeeper [19], NormFinder [20], and geNorm [21]. Gene expression stability was assessed in bud tissues at four post-inoculation time points, corresponding to early stages of infection, in two distinct genotypes: a commercial sugarcane cultivar (smut-intermediate resistant, IACSP02-1064) and commercial energy cane (smut-susceptible, Vertix 10) following *S. scitamineum* inoculation.

The selected reference genes were further evaluated by analyzing the expression profile of *Saccharum SPL19* (*SQUAMOSA promoter-binding protein-like 19*), which belongs to the SQUAMOSA PROMOTER-BINDING PROTEIN-LIKE (SPL) family of transcription factors, whose members are involved in the regulation of plant developmental processes [22]. In the intermediate smut-resistant cultivar RB925345, sugarcane *SPL* genes were responsive to smut infection [23]. The expression profile of *ScSPL19* was evaluated using the most and least stable reference gene pairs identified in this study.

Our results revealed stable expression profiles and highlighted potential genotype-specific variations during pathogen colonization, providing a reliable interpretation of gene expression data within the *Saccharum*–smut pathosystem. We further observed differential temporal regulation of *ScSPL19* during infection, with earlier repression in the intermediate-resistant cultivar and delayed repression in the susceptible genotype, suggesting a potential association between *ScSPL19* regulation and the contrasting responses to *S. scitamineum* infection.

## 2. Results

### 2.1. Primer Efficiencies and Specificity

Amplification efficiency (E), primer concentration, correlation coefficient (R^2^), cycle threshold (Ct) mean, and standard deviation (SD) for the candidate reference gene primers varied across the evaluated genotypes. The amplification efficiencies obtained during the initial primer concentration optimization are presented in Supplementary Table S1. The primer concentrations established based on the preliminary optimization were subsequently used in the reference gene stability analysis. Under these final assay conditions, all candidate reference gene primers showed amplification efficiencies within the acceptable range (>90%), although differences in efficiency were observed among genes and between genotypes (Table 1). The differences are detailed below.

In IACSP02-1064 cultivar, the amplification efficiency was ranked as follows: *ACAD* (*Acyl-CoA dehydrogenase family member 10*) > *UBQ1 *(*Ubiquitin 1*) > *ACT* (*Actin*) > *25SrRNA2* (*25S ribosomal RNA 2*) > *GAPDH *(*Glyceraldehyde-3-phosphate dehydrogenase*) > *25SrRNA1 *(*25S ribosomal RNA 1*) > *SARMp1 *(*Serine/arginine repetitive matrix protein 1*) > *UBQ2 *(*Ubiquitin 2*). In contrast, the ranking in Vertix 10 genotype was *ACAD* > *GAPDH* > *SARMp1* > *UBQ1* > *25SrRNA2* > *ACT* > *25SrRNA1* > *UBQ2*. Across both genotypes, primer efficiencies ranged from 91% to 102%, fitting within the acceptable range for RT-qPCR. High correlation coefficients (R^2^ > 0.99) were observed for most genes, except for *SARMp1* in IACSP02-1064 (Table 1).

In IACSP02-1064, the optimal efficiencies were obtained at a primer concentration of 1.2 μM for *UBQ2*, 1.0 μM for *ACAD*, *ACT*, *25SrRNA1*, *25SrRNA2*, and *GAPDH*, and 0.25 μM for *SARMp1* and *UBQ1*. In Vertix 10, the corresponding concentrations were 1.2 μM for *UBQ2*, 1.0 μM for *ACAD*, *ACT*, *25SrRNA2*, and *GAPDH*, 0.8 μM for *SARMp1* and *UBQ1*, and 0.7 μM for *25SrRNA1*. The E value, R^2^, Ct mean, and SD for each gene are presented in Table 1. Primer specificity was also evaluated through dissociation (melting) curve analysis. The absence of non-specific amplification products and primer dimers was demonstrated using melting curve analysis. In each case, a single fluorescence peak corresponding to a specific PCR product was observed (Figure S1).

### 2.2. Stability of Candidate Reference Genes

The expression stability of the eight candidate genes was independently assessed using three algorithms (Table 2). For IACSP02-1064 cultivar, both BestKeeper and NormFinder identified *ACAD* as the most stable gene, whereas geNorm ranked *GAPDH*. In the Vertix 10 cultivar, BestKeeper and NormFinder identified *GAPDH* as the most stable candidate, while geNorm prioritized *ACAD*. Despite differences in ranking among the algorithms, *GAPDH* and *UBQ2* were consistently classified among the most stable genes in this genotype. As observed for IACSP02-1064, greater variability was detected among the algorithms in identifying the least stable candidates compared to the most stable ones.

### 2.3. Optimal Number of Reference Genes

**Figure 1:**
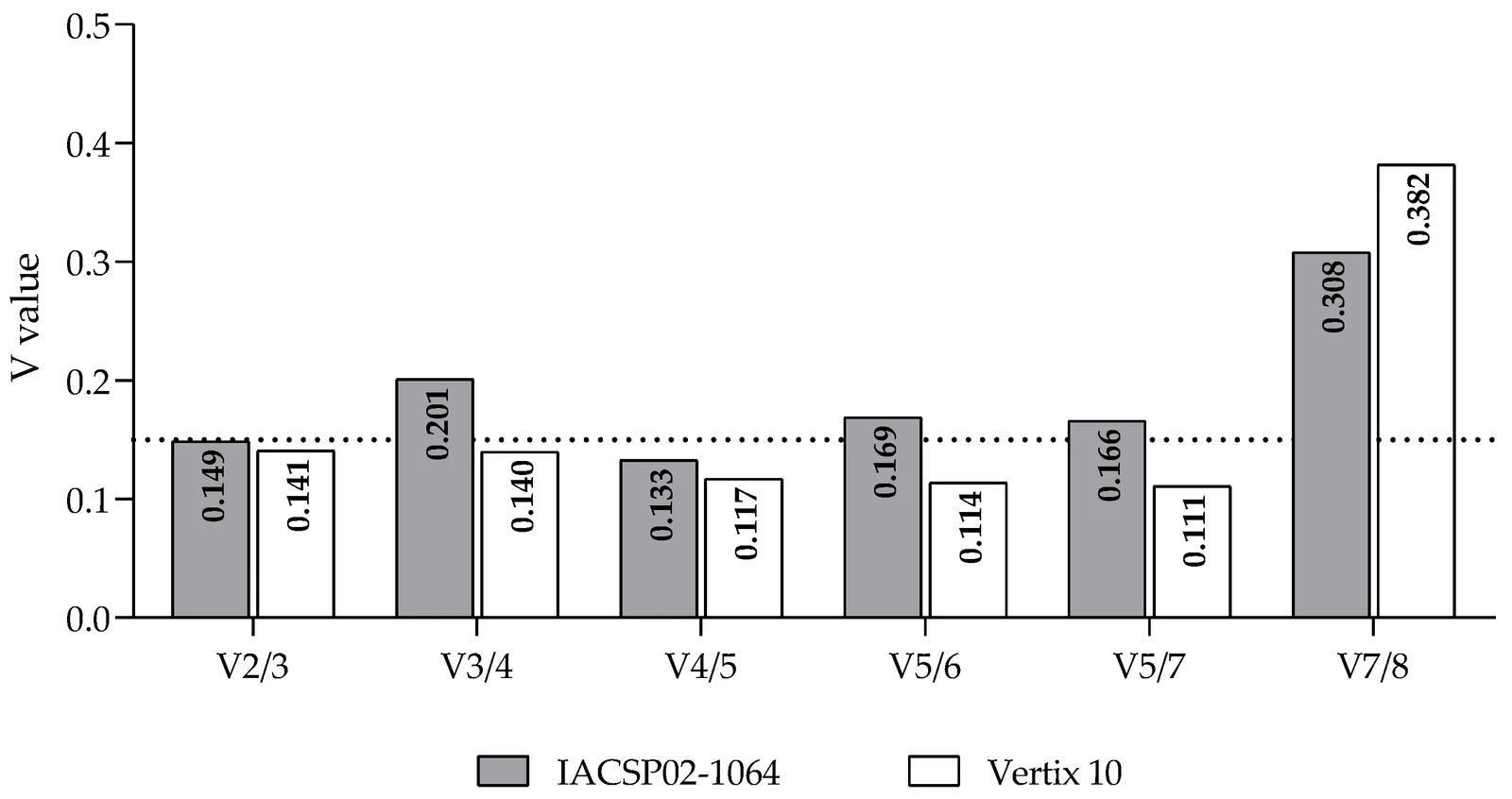
Determination of the optimal number of reference genes for normalization. Pairwise variation (Vn/Vn + 1) values across six pairwise comparisons (V2/3 to V7/8) for the two genotypes, IACSP02-1064 and Vertix 10. A value of V < 0.15, indicated by the horizontal dashed line, was used as the cut-off [21].

Pairwise variation analysis (Vn/Vn + 1) performed using geNorm showed that the V2/3 values were below the threshold of V < 0.15 proposed by Vandesompele et al. [21] for both genotypes. These results indicated that the addition of a third reference gene did not substantially improve normalization, as the use of two reference genes was sufficient for reliable normalization under *S. scitamineum* inoculation conditions (Figure 1).

The NormFinder algorithm identified the *ACAD*/*UBQ2* combination as the optimal pair for IACSP02-1064 cultivar under smut infection followed by *ACAD*/*GAPDH* (Table 3). In contrast, geNorm identified *ACAD*/*GAPDH* as the most stable pair for IACSP02-1064 and *GAPDH*/*UBQ2* as the most stable combination for the Vertix 10 genotype (Table 3).

### 2.4. Evaluation of Reference Gene Performance Using ScSPL19

To evaluate the impact of reference gene selection on target-gene normalization, the expression of the target gene *ScSPL19* was normalized using the common high-performing reference gene pair (*GAPDH*/*ACAD*) and least stable gene pair (*25SrRNA2*/*ACT*). This approach was adopted to allow direct comparison of *ScSPL19* expression profiles between IACSP02-1064 and Vertix 10 using the same normalization strategy. Expression levels were measured at 12, 24, 48, and 120 hpi in *S. scitamineum*-inoculated versus mock-inoculated treatments across the two *Saccharum* cultivars (IACSP02-1064 and Vertix 10). All evaluations included three biological and three technical replicates per condition.

**Figure 2:**
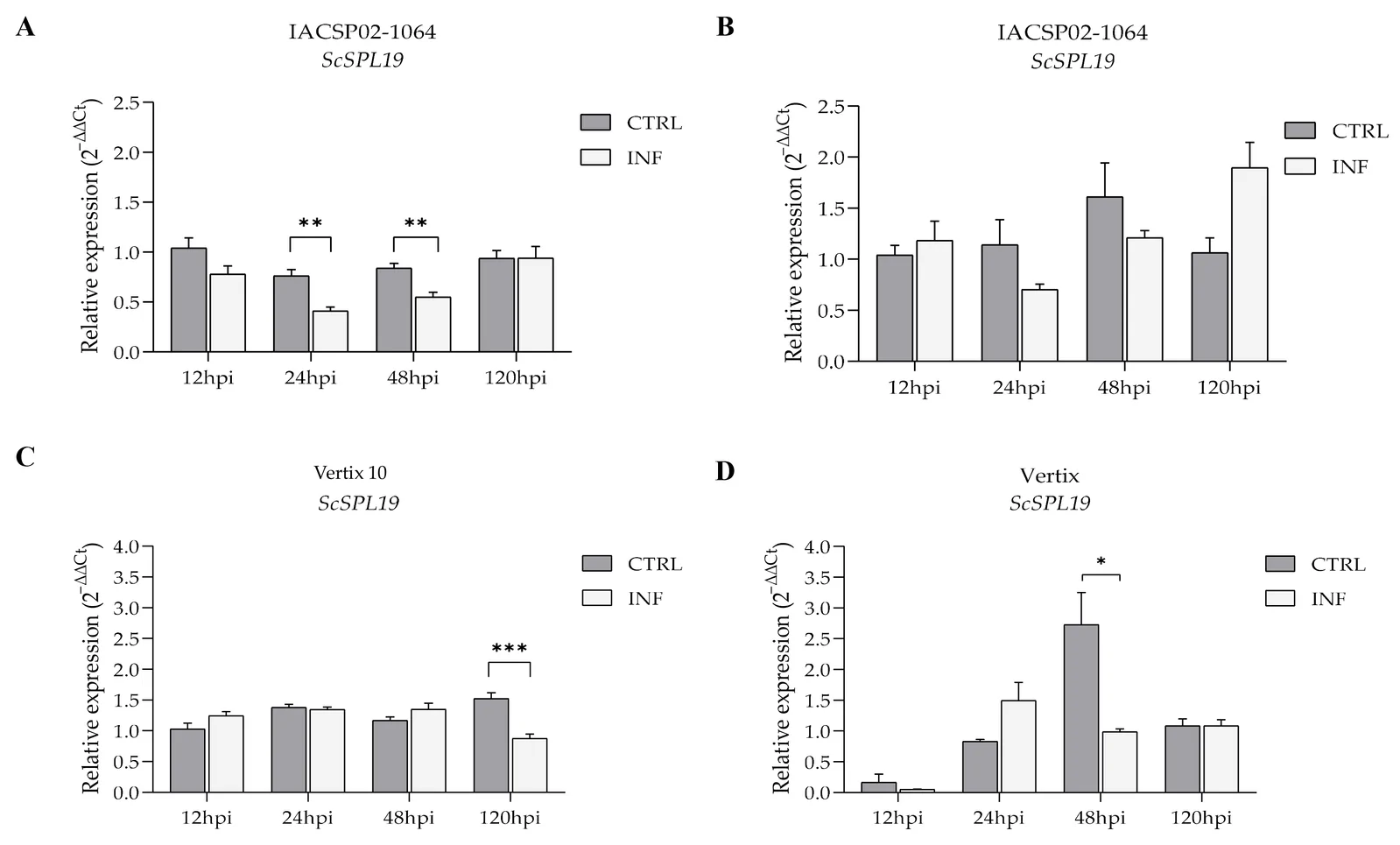
Relative expression of target gene *ScSPL19* (*SQUAMOSA promoter-binding protein-like 19* from *Saccharum* spp.) normalized using the most and least stable reference gene pairs. Error bars represent the standard error of the mean (SEM) based on three biological replicates. (**A**) *ScSPL19* expression in IACSP02-1064 normalized with the most stable gene pair (*GAPDH*/*ACAD*). (**B**) *ScSPL19* expression in IACSP02-1064 normalized with the least stable gene pair (*25SrRNA2*/*ACT*). (**C**) *ScSPL19* expression in Vertix 10 normalized with the most stable gene pair (*GAPDH*/*ACAD*). (**D**) *ScSPL19* expression in Vertix 10 normalized with the least stable gene pair (*25SrRNA2*/*ACT*). Statistical significance was determined using two-way ANOVA and mixed-effects analysis for parametric samples, followed by Bonferroni’s post-hoc test. Asterisks indicate statistically significant differences: *p* < 0.001 (***), *p* < 0.01 (**), and *p* < 0.05 (*). Abbreviations: hpi, hours post-inoculation (12, 24, 48, and 120 hpi); CTRL, mock controls; INF, inoculated samples.

In cultivar IACSP02-1064, *ScSPL19* was downregulated in the inoculated group at 24 and 48 hpi when normalized to the most stable gene pair, *GAPDH*/*ACAD* (Figure 2A). In contrast, using the least stable gene pair, *25SrRNA2/ACT*, resulted in no statistical significance in expression between the treatments (Figure 2B). Additionally, cultivar Vertix 10 exhibited *ScSPL19* downregulation in the inoculated group at 120 hpi when normalized with the most stable gene pair, *GAPDH*/*ACAD* (Figure 2C). However, using the least stable gene pair, *25SrRNA2*/*ACT*, the results indicated a downregulation in the inoculated group at 48 hpi (Figure 2D). These contrasting expression profiles demonstrate that reference gene selection can affect both the timing and detection of target gene responses to smut infection.

## 3. Discussion

The identification of stable reference genes is a critical prerequisite for accurate RT-qPCR normalization, particularly in plant–pathogen interactions characterized by dynamic changes in gene expression following infection. In this study, we evaluated the stability of eight candidate reference genes in the meristematic buds of two *Saccharum* genotypes, the sugarcane cultivar IACSP02-1064 and the energy cane genotype Vertix 10, during the early stages of infection by *S. scitamineum*. These genotypes differ in their susceptibility to the smut fungus, with IACSP02-1064 showing intermediate resistance and Vertix 10 being susceptible.

The candidate reference genes evaluated in this study were selected based on previous reports of their stability in *Saccharum* spp. hybrids under biotic and abiotic stress conditions [13,14,15,17,18,24,25,26]. In their study of the sugarcane–*S. scitamineum* interaction, Huang et al. [15] ranked *ACAD* and *SARMp1* as the most stable reference genes according to the comprehensive ranking. In contrast, commonly used reference genes such as *elongation factor 1-alpha* (*eEF1a*) and *cullin* (*CUL*) did not rank among the most stable candidates in that study, despite their reported use under other experimental conditions [18]. These findings provided a rationale for candidate selection while highlighting the need to validate reference gene stability under the specific conditions investigated.

The temporal resolution of this study, encompassing four early infection time points (12, 24, 48, and 120 hpi), is particularly relevant because these stages span the establishment of the pathogen and the activation of the host defense responses, during which extensive transcriptional reprogramming is expected [27]. Consequently, identifying reference genes that remain stably expressed throughout this dynamic period is essential for accurate RT-qPCR normalization. Moreover, evaluating two distinct *Saccharum* genotypes across this infection timeline supports the use of the evaluated reference genes in comparative gene expression studies aimed at investigating the molecular basis of smut resistance.

Using three complementary statistical algorithms (NormFinder (R implementation, version dated January 5, 2015), geNorm v3.5, and BestKeeper v.1), we consistently identified *GAPDH* and *ACAD* among the most stable reference genes across the experimental conditions analyzed. The stable performance of *ACAD* and *GAPDH* across the evaluated conditions is particularly noteworthy given the dynamic nature of the early stages of *S. scitamineum* infection. Although minor differences were observed among the individual rankings generated by NormFinder, geNorm, and BestKeeper, all three algorithms consistently identified these genes among the most stable candidates across both genotypes (Table 2 and Table 3). ACAD participates in fatty acid β-oxidation, a conserved metabolic process essential for cellular energy metabolism [28]. Its stable expression during smut infection, consistent with previous reports [14,15], further supports its suitability as a reliable reference gene for RT-qPCR normalization in sugarcane and energy cane buds infected with *S. scitamineum*.

GAPDH is a conserved key enzyme in glycolysis, an essential metabolic pathway required for cellular energy production [29]. Because its expression remains stable across several biological conditions [18,30], *GAPDH* has been widely used as a reference gene. However, its transcription has been reported to vary under certain stress conditions [14,15,31], as it regulates metabolic pathways to provide the substrates required for energy production and the synthesis of defense-related compounds [29]. Nevertheless, we show that *GAPDH* exhibited stable expression across the evaluated infection conditions, supporting its suitability as a reference gene for RT-qPCR normalization under the conditions evaluated.

Another important observation is the relatively low stability of some traditionally used housekeeping genes, such as *UBQ1* (Table 2). Although *UBQ1* is widely used as an internal control in plant molecular studies [13,24], our results indicate that its expression may vary substantially in bud tissues during the early stages of smut infection. Similar instability of *UBQ1* has been reported in other sugarcane cultivars (IACSP95-5000 and IAC91-1099), when inoculated with a severe strain of the sugarcane mosaic virus (SCMV) [17]. On the other hand, *UBQ2*, a member of the same gene family, proved to be highly stable under our experimental conditions (Table 2 and Table 3). These differences in the stability of ubiquitin-related genes may reflect their distinct regulation in response to specific tissues, developmental stages, and stress conditions. Ubiquitin-related processes are involved in the regulation of protein stability and signaling and may therefore contribute to the dynamic regulation of metabolic and immune responses during stress [32,33].

The complex polyploid genome of *Saccharum* spp. may contribute to substantial transcriptional plasticity and genotype-dependent differences in gene expression. In this context, different genotypes may exhibit distinct patterns of gene regulation, including differential expression of gene copies under stress conditions, potentially influenced by mechanisms such as DNA methylation [34,35]. Such genotype-dependent transcriptional responses may affect the stability of candidate reference genes and, consequently, the selection of the most suitable genes for RT-qPCR normalization. This may explain the differences in reference gene stability observed between our study and previous reports using the sugarcane cultivars NCo376 and YC71-374 [15] and the energy cane cultivars Vertix 1 and Vertix 2 [14]. These differences may also be influenced by experimental factors, including the fungal isolate, inoculum concentration, and sampling strategy.

To assess the suitability of the selected reference gene for target gene normalization, we analyzed the expression of *ScSPL19* using the most stable reference gene pair (*GAPDH*/*ACAD*) and the least stable pair (*25SrRNA2*/*ACT*). When normalized using *GAPDH*/*ACAD*, *ScSPL19* was downregulated at 24 and 48 hpi in the intermediate smut-resistant cultivar IACSP02-1064, whereas in the susceptible cultivar Vertix 10, downregulation was detected only at 120 hpi (Figure 2A,C). This distinct temporal pattern suggests a more rapid modulation of *ScSPL19* expression in the intermediately resistant genotype in response to pathogen infection. Given that SPL proteins are involved in the regulation of plant developmental processes, the early repression of *ScSPL19* in IACSP02-1064 could potentially be associated with the modulation of developmental programs in response to pathogen challenge, although this possibility remains to be experimentally investigated. Similar interactions between pathogen responses and developmental regulations have been reported in *Arabidopsis thaliana*, where the inhibition of flowering during *Fusarium oxysporum* infection was proposed as a mechanism to redirect resources toward defense [36].

In IACSP02-1064, normalization with the least stable reference gene pair (*25SrRNA2*/*ACT*) revealed no significant difference in *ScSPL19* expression at any time point (Figure 2B). Furthermore, in Vertix 10, normalization with this same unstable combination resulted in *ScSPL19* downregulation at 48 hpi (Figure 2D). Transcription factors, such as the one investigated here, play crucial roles in regulating gene expression in response to environmental, biotic, and abiotic stimuli [37]. Because they can regulate broad transcriptional and metabolic responses even at relatively low expression levels, accurately detecting subtle changes in their transcripts requires precise normalization. In our study, the use of the most stable reference gene combination resulted in a distinct and temporally resolved expression profile, whereas normalization with the least stable combination altered the detection of treatment-associated differences. Ultimately, these contrasting patterns reinforce how the use of less stable reference genes can substantially alter the interpretation of target gene expression, underscoring the necessity of reference gene validation to accurately assess transcriptional responses during *S. scitamineum* infection.

Overall, our results demonstrate that reference gene validation is essential for reliable RT-qPCR normalization in the *Saccharum*–smut pathosystem, particularly across different genotypes and infection stages. The *ACAD*/*GAPDH* combination emerged among the most stable reference genes in both IACSP02-1064 and Vertix 10, supporting its suitability for normalization under the conditions evaluated. The evaluation of the reference gene rankings using *ScSPL19* further demonstrated that reference gene selection can substantially influence the detection and interpretation of genotype- and time-dependent transcriptional responses to *S. scitamineum* infection. By identifying suitable reference gene combinations for sugarcane and energy cane bud tissues across early infection stages, this study provides a basis for accurate relative gene expression analyses and for investigating genotype-specific responses to smut infection. These findings provide a useful framework for RT-qPCR normalization in *Saccharum* bud tissues under the evaluated *S. scitamineum* infection conditions.

## 4. Materials and Methods

### 4.1. Biological Material and Experimental Conditions

To investigate gene expression responses to smut infection, two genotypes with contrasting susceptibility to smut were selected. Sugarcane cultivar IACSP02-1064 (intermediate-resistant) was provided by the Agronomic Institute (IAC), Ribeirao Preto, Brazil, and Vertix 10 (smut-susceptible) by Nuseed Brazil. To ensure the use of disease-free material, the buds were subjected to thermal treatment by immersion in water at 52 °C for 30 min [38]. After disinfection, the bud sets were placed in cell trays with moist cotton and incubated at 30 °C until the time of inoculation.

*S. scitamineum* teliospores were obtained from diseased plants in a previously infected experimental field plot at the Sugarcane Center in Ribeirão Preto, São Paulo, Brazil. Prior to storage, teliospore viability was assessed (germination rate > 90%), and the samples were dehydrated using silica gel(Quimpack, Concórdia SC, BR) and stored at 4 °C until use. Following the protocol adapted from Peters et al. [39], inoculation was performed by applying a 20 µL drop of spore suspension containing 5 × 10^6^ spores mL^−1^ in 0.85% NaCl and 0.01% (*v*/*v*) Tween-20 to the bud base after causing a needle injury. Mock controls received the same solution (0.85% NaCl + 0.01% Tween-20) without spores. After inoculation, both inoculated and control treatments were maintained in a BOD incubator at 30 °C in the dark. Three independent biological replicates, each consisting of 9 buds (totaling 27 buds per treatment), were collected at 12, 24, 48, and 120 h post-inoculation (hpi). Collected samples were immediately stored in sterile 50 mL tubes at −80 °C for subsequent molecular analysis.

### 4.2. Total RNA Extraction and cDNA Synthesis

Total RNA was extracted using TRIzol (Sigma-Aldrich, Saint Louis, MO, USA) followed by purification with the PureLink™ RNA Mini Kit (Invitrogen, Waltham, MA, USA). RNA concentration and purity were measured using NanoDrop 2000 (Thermo Fisher Scientific, Waltham, MA, USA), with A260/A280 ratios consistently ranging from 2 to 2.2 (Table S2). RNA integrity was further evaluated by agarose gel electrophoresis. Before cDNA synthesis, residual genomic DNA was removed from the total RNA samples using DNase I Amplification Grade (Sigma-Aldrich). First-strand cDNA synthesis was performed using the GoScript™ Reverse Transcriptase kit (Promega, Fitchburg, WI, USA) according to the manufacturer’s instructions. Reactions were carried out using 1 μg of total RNA as input and oligo (dT) primers. All resulting cDNA samples were diluted 1:10 prior to downstream applications.

For each genotype, cDNA from the three biological replicates was pooled for each specific treatment and time point. This resulted in eight distinct pooled cDNA samples per genotype, corresponding to the inoculated and mock-treated conditions at 12, 24, 48, and 120 hpi. Each pooled sample therefore represented cDNA obtained from the three biological replicates, comprising a total of 27 buds per treatment and time point. The same pooled samples, prepared at a single 1:10 dilution, were used for primer efficiency determination and subsequent reference gene stability analyses. Because amplification efficiencies were derived directly from the individual amplification curves using LinRegPCR 7.5 software [40], a standard curve of serial dilutions was not required. The target gene expression assessment using the selected reference genes was performed using the three biological replicates individually, without pooling, with three technical replicates per biological sample.

### 4.3. Selection of Reference Genes

A total of eight candidate reference genes from *Saccharum* spp. (*ACT*, *GAPDH*, *UBQ1*, *UBQ2*, *25SrRNA1*, *25SrRNA2*, *ACAD*, and *SARMp1*) were selected based on their previously reported stability in *Saccharum* spp. hybrids under abiotic and biotic stresses [13,14,15,17,18,24,25,26]. The selection of *ACAD* and *SARMp1* was further supported by Huang et al. [15], who identified both genes as the most stable reference genes according to the comprehensive ranking in the sugarcane–*S. scitamineum* interaction system. The other candidates were selected based on previous reports of their stability in *Saccharum* spp. and their use in our laboratory under related experimental conditions. Primer sequences for these candidates were obtained from the literature and are listed in Table 4.

An initial primer optimization step was performed to establish suitable primer concentrations for the subsequent RT-qPCR assays. Pooled cDNA samples were diluted 1:10 and analyzed in two technical replicates per condition. Primer concentrations of 0.2, 0.4, and 0.8 μM were initially evaluated. For primer pairs showing suboptimal amplification efficiency or amplification profiles, higher primer concentrations (1.0 and/or 1.2 μM) were subsequently tested (Table S1). Rather than using a standard curve of serial dilutions, individual amplification efficiencies (E) and the squared correlation coefficient (R^2^) were determined directly from the exponential phase of individual amplification curves using LinRegPCR 7.5 software [40]. Finally, the optimal concentration for subsequent runs was selected based on the resulting primer efficiencies and melting curve profiles. Melting curves obtained under the final primer concentrations used for the reference gene stability analysis are presented in Figure S1.

The stability of the eight candidate reference genes was evaluated using three complementary statistical algorithms, NormFinder (R package version dated 5 January 2015), BestKeeper v.1, and geNorm v3.5. For each gene, the mean Ct value obtained from technical duplicates was used for subsequent analyses. The results obtained from these complementary approaches were used to identify the most stable reference genes under experimental conditions.

### 4.4. Effect of Reference Gene Selection on ScSPL19 Expression Analysis

The target gene *ScSPL19* was used to assess the suitability of the selected reference genes for target-gene normalization. In this analysis, three biological replicates were analyzed in technical triplicates, and the mean Ct value of the technical replicates was used for subsequent analysis. The web tool Primer3Plus (http://www.primer3plus.com (accessed on 2 April 2023)) was used to design the primers for this gene adopting as conditions: primer length ranging between 17 and 22 bp, GC content between 35–65 (%), and melting temperature (Tm) between 59 °C and 65 °C with an amplicon ranging between 100 and 250 bp. The web tool NetPrimer (www.premierbiosoft.com/netprimer (accessed on 2 April 2023)) was used to analyze primer quality for secondary structure formation.

### 4.5. RT-qPCR Conditions

RT-qPCR reactions were performed using the Applied Biosystems StepOnePlus System (Applied Biosystems, Foster City, CA, USA). The reactions were conducted in a final volume of 10 μL containing 5 μL of SYBR Green Super Mix (Promega), 3 μL of cDNA (1:10) and primer pairs at their optimized concentrations. Thermal cycling conditions consisted of an initial denaturation at 95 °C for 2 min, followed by 40 cycles at 95 °C for 15 s and 60 °C for 60 s. After the amplification cycles, the specificity of the amplicons was analyzed through the dissociation curve profiles (melting curve).

### 4.6. Statistical Analysis

The *ScSPL19* relative expression levels were calculated using the 2^−ΔΔCt^ method [41] and expressed as fold change relative to the corresponding control samples. For the ΔCt calculation, the geometric mean of the Ct values obtained for the two reference genes was used for normalization. To assess the impact of reference gene selection on the observed *ScSPL19* expression pattern, the results obtained using the most stable reference gene pair (*GAPDH*/*ACAD*) were compared with those obtained using the least stable pair (*25SrRNA2*/*ACT*). The data were expressed as the mean and standard error of the mean (SEM) calculated from biological replicates. For the statistical analysis, two-way ANOVA and mixed-effects analysis were employed for parametric data, followed by the Bonferroni post-hoc test, with *p* ≤ 0.05 considered statistically significant. GraphPad Prism Software v.8.0.2 was used to perform the statistical analyses, as well as for graph construction and image generation.

## Figures and Tables

**Table 1:** Candidate reference genes and primer concentrations used for the reference gene stability analysis.

Cultivar	Gene Name	Primer Concentration (µM)	*E* (%)	*R* ^2^	Ct Mean	SD
IACSP02-1064	*25SrRNA1*	1	96	0.9998	20.62	0.503
	*25SrRNA2*	1	98.8	0.9998	21.13	0.348
	*ACAD*	1	99.9	0.9997	24.87	0.890
	*ACT*	1	98.8	0.9998	26.8	3.119
	*GAPDH*	1	96	0.9995	16.44	0.778
	*SARMp1*	0.25	95.3	0.9579	28.55	1.697
	*UBQ1*	0.25	98.9	0.9998	30.59	1.239
	*UBQ2*	1.2	91.2	0.9998	18.58	0.924
Vertix 10	*25SrRNA1*	0.7	98.2	0.9999	20.57	0.606
	*25SrRNA2*	1	100.2	0.9998	20.79	0.823
	*ACAD*	1	102	0.9997	23.18	0.681
	*ACT*	1	99.7	0.9998	25.91	3.365
	*GAPDH*	1	101.4	0.9996	15.9	0.815
	*SARMp1*	0.8	101.3	0.9997	28.85	0.896
	*UBQ1*	0.8	100.8	0.9998	29.86	0.797
	*UBQ2*	1.2	92.8	0.9999	16.49	0.857

Efficiency (*E*), correlation coefficient squared (*R*^2^), cycle threshold (Ct) mean and standard deviation (SD), in pooled bud cDNA samples of IACSP02-1064 and Vertix 10 cultivars.

**Table 2:** Expression stability analysis of eight candidate reference genes in the buds of contrasting sugarcane and energy cane genotypes under *S. scitamineum* inoculation. The most stable genes identified by each algorithm are highlighted in bold. SD: standard deviation of technical duplicates.

		NormFinder	geNorm	BestKeeper		
Cultivar	Gene	Stability	M < 1.5	Stability	SD	*p*-Value
IACSP02-1064	*25SrRNA1*	0.92	1.261	0.248	0.381	0.353
	*25SrRNA2*	0.97	1.224	0.261	0.266	0.330
	*ACAD*	**0.36**	0.977	**0.949**	0.690	0.001
	*ACT*	2.04	2.524	0.925	2.632	0.001
	*GAPDH*	0.38	**0.974**	0.942	0.587	0.001
	*SARMp1*	0.83	1.383	0.898	1.400	0.001
	*UBQ1*	0.90	1.286	0.697	0.980	0.003
	*UBQ2*	0.56	1.091	0.848	0.702	0.001
Vertix 10	*25SrRNA1*	0.87	1.116	0.311	0.479	0.243
	*25SrRNA2*	0.94	1.256	0.202	0.681	0.454
	*ACAD*	0.42	**0.908**	0.839	0.517	0.001
	*ACT*	2.30	3.072	0.772	2.593	0.001
	*GAPDH*	**0.26**	0.989	**0.937**	0.587	0.001
	*SARMp1*	0.40	1.040	0.856	0.719	0.001
	*UBQ1*	0.59	0.965	0.768	0.660	0.001
	*UBQ2*	0.36	0.957	0.904	0.596	0.001

**Table 3:** Optimal reference gene pairs among eight candidate genes in bud tissues of contrasting sugarcane and energy cane genotypes under *S. scitamineum* inoculation.

	NormFinder			geNorm	
Cultivar	Gene 1	Gene 2	Stability	Gene 1	Gene 2
IACSP02-1064	*ACAD*	*UBQ2*	0.32		
	*ACAD*	*GAPDH*	0.33	*ACAD*	*GAPDH*
	*ACAD*	*SARMp1*	0.49		
Vertix 10	*ACAD*	*GAPDH*	0.31		
	*ACAD*	*SARMp1*	0.36	*GAPDH*	*UBQ2*
	*ACAD*	*UBQ2*	0.4		

**Table 4:** List of reference genes tested in this study.

Gene	Gene Description	Primer Sequence (5′-3′)	Amplicon Size (bp)	Reference
*ACAD*	*Acyl-CoA dehydrogenase family member 10*	F: CGTGGCATGGATCTGATGGT R: AGCCTGCTCCAGTTCAATCC	120	[15]
*UBQ1*	*Ubiquitin 1*	F: AGCCTCAGACCAGATTCCAA R: AATCGCTGTCGAACTACTTGC	110	[13]
*ACT*	*Actin*	F: CTCAACCCCAAGGCTAACAG R: GGCATGAGGAAGGGCATAA	195	[16]
*25SrRNA2*	*25S ribosomal RNA*	F: GCAGCCAAGCGTTCATAGC R: CCTATTGGTGGGTGAACAATCC	108	[13]
*GAPDH*	*Glyceraldehyde-3phosphate dehydrogenase*	F: TTGGTTTCCACTGACTTCGTT R: CTGTAGCCCCACTCGTTGT	122	[16]
*25SrRNA1*	*25S ribosomal RNA*	F: ATAACCGCATCAGGTCTCCAAG R: CCTCAGAGCCAATCCTTTTCC	110	[16]
*SARMp1*	*Serine/arginine repetitive matrix protein 1*	F: TGGACTTGGTCAGTTGGAAACA R: TGTTCCTGAAGCCTATGTTGCT	102	[15]
*UBQ2*	*Ubiquitin 2*	F: CTTCTTCTGTCCCTCCGATG R: TCCAACCAAACTGCTGCTC	158	[13]

## Data Availability

Data are contained within this article and the Supplementary Materials.

## Supplementary Materials

The following supporting information can be downloaded at: https://www.mdpi.com/article/doi/s1/.

## Abbreviations

The following abbreviations are used in this manuscript:

RT-qPCR	Reverse Transcription–Quantitative Polymerase Chain Reaction
hpi	Hours post-inoculation
cDNA	Complementary DNA
*ACT*	*Actin*
*GAPDH*	*Glyceraldehyde-3-phosphate dehydrogenase*
*UBQ1*	*Ubiquitin 1*
*UBQ2*	*Ubiquitin 2*
*25SrRNA1*	*25S ribosomal RNA 1*
*25SrRNA2*	*25S ribosomal RNA 2*
*ACAD*	*Acyl-CoA dehydrogenase family member 10*
*SARMp1*	*Serine/arginine repetitive matrix protein 1*
E	Amplification efficiency
R^2^	Correlation coefficient square
Ct	Cycle threshold
SD	Standard deviation
V	Pairwise variation
IAC	Agronomic Institute (Instituto Agronômico)
SCMV	Sugarcane Mosaic Virus
BOD	Biochemical oxygen demand (incubator)
