# Supplementary material for: Validation of Reference Genes for RT-qPCR Normalization in Saccharum Genotypes During Smut Infection
Source: Int J Mol Sci. 2026 Oct 22;27(21):9393. doi: 10.3390/ijms27219393 (PMC13763242; doi:10.3390/ijms27219393)
Supplement: Supplementary file 1 [file ijms-27-09393-s001.zip › ijms-4574389-supplementary.pdf]

SUPPLEMENTARY MATERIALS

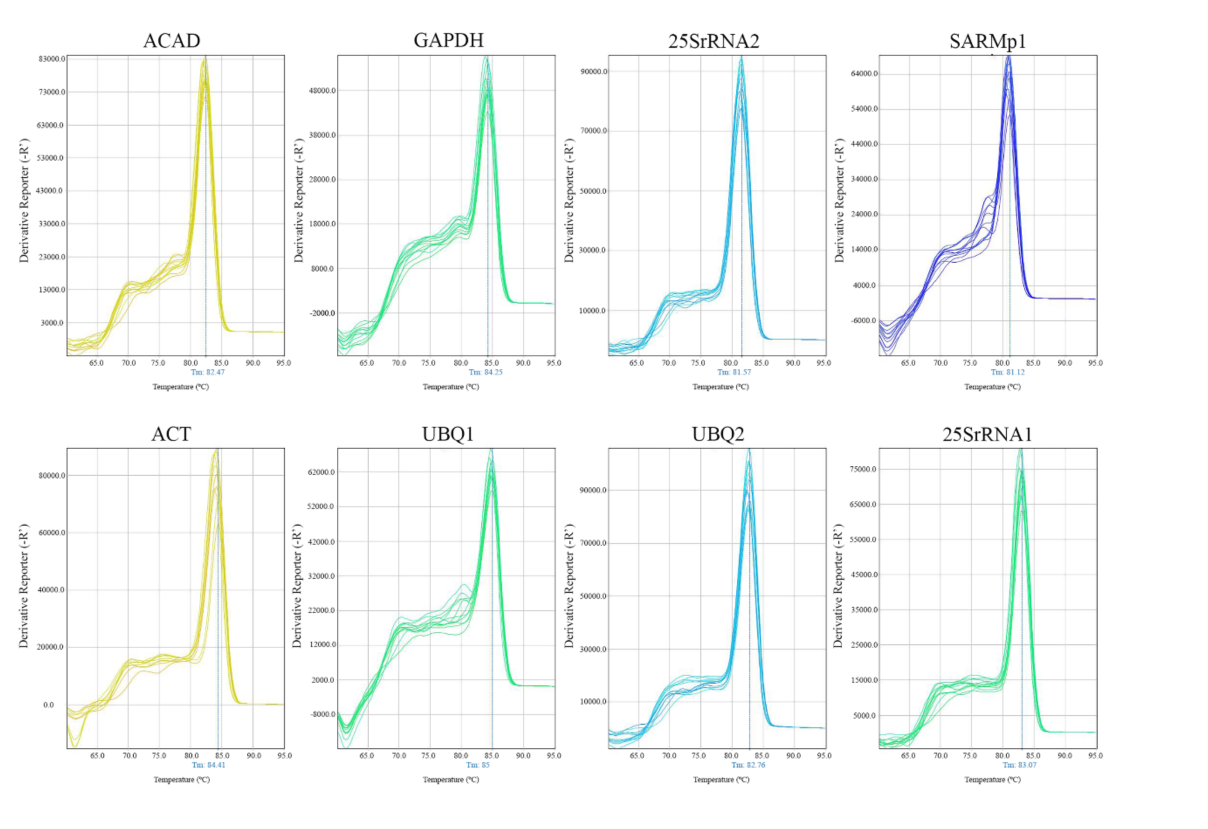

Figure S1. **Dissociation curve generated by RT-qPCR reactions for endogenous control.** Single peaks represent high-quality primers.

Table S1. Amplification efficiencies obtained during the initial primer concentration optimization in IACSP02-1064 and Vertix 10 (IACSP02-1064/Vertix 10).

Gene	0.2 µM	0.4 µM	0.8 µM	1.0 µM	1.2 µM
ACT	76.7 / 76.9	89.2 / 78.2	88.6 / 84.8	83.7 / 90.0	– / –
ACAD	80.5 / 77.6	87.2 / 86.0	91.4 / 87.7	92.9 / 90.7	– / –
UBQ1	84.1 / 81.5	89.6 / 88.1	94.4 / 93.2	– / –	– / –
UBQ2	70.2 / 69.2	20.9 / 25.5	77.4 / 79.5	– / –	73.3 / 83.6
GAPDH	57.9 / 63.6	74.1 / 61.1	64.1 / 68.0	76.4 / 90.1	– / –
SARMp1	83.1 / 81.8	89.8 / 91.7	98.8 / 97.5	– / –	– / –
25SrRNA1	69.2 / 68.4	82.2 / 78.6	83.9 / 84.0	84.0 / 101.4	– / –
25SrRNA2	78.0 / 81.4	83.1 / 86.4	87.0 / 90.0	93.3 / 96.4	– / –

* Primer concentrations of 0.2, 0.4, and 0.8 µM were initially evaluated. Higher concentrations (1.0 and/or 1.2 µM) were subsequently tested when required based on the amplification performance.

Table S2. RNA concentration and 260/280 OD ratios.

Sample ID	Nucleic Acid Conc.	Unit	260/280
IACSP02-1064_CTRL(12hpi1)	1661.5	ng/µl	2.11

IACSP02-1064_CTRL(12hpi2)	1324.3	ng/µl	2.12
IACSP02-1064_CTRL(12hpi3)	1220	ng/µl	2.12
IACSP02-1064_CTRL(24hpi1)	1280.7	ng/µl	2.12
IACSP02-1064_CTRL(24hpi2)	1093.9	ng/µl	2.08
IACSP02-1064_CTRL(24hpi3)	1025.7	ng/µl	2.09
IACSP02-1064_CTRL(48hpi1)	1332.1	ng/µl	2.12
IACSP02-1064_CTRL(48hpi2)	1316.8	ng/µl	2.11
IACSP02-1064_CTRL(48hpi3)	1291.8	ng/µl	2.07
IACSP02-1064_CTRL(120hpi1)	1575.9	ng/µl	2.09
IACSP02-1064_CTRL(120hpi2)	1114.5	ng/µl	2.13
IACSP02-1064_CTRL(120hpi3)	1616.1	ng/µl	2.04
IACSP02-1064_INF(12hpi1)	1048	ng/µl	2.06
IACSP02-1064_INF(12hpi2)	1158.8	ng/µl	2.15
IACSP02-1064_INF(12hpi3)	871.3	ng/µl	2.12
IACSP02-1064_INF(24hpi1)	982.9	ng/µl	2.13
IACSP02-1064_INF(24hpi2)	1393.3	ng/µl	2.08
IACSP02-1064_INF(24hpi3)	1058.5	ng/µl	2.07
IACSP02-1064_INF(48hpi1)	1185.4	ng/µl	2.11
IACSP02-1064_INF(48hpi2)	1375.1	ng/µl	2.13
IACSP02-1064_INF(48hpi3)	1380.9	ng/µl	2.11
IACSP02-1064_INF(120hpi1)	1489	ng/µl	2.12
IACSP02-1064_INF(120hpi2)	1435.3	ng/µl	2.12
IACSP02-1064_INF(120hpi3)	1274.4	ng/µl	2.08
Vertix10_CTRL(12hpi1)	998.8	ng/µl	2.13
Vertix10_CTRL(12hpi2)	662.4	ng/µl	2.14
Vertix10_CTRL(12hpi3)	819.4	ng/µl	2.15
Vertix10_CTRL(24hpi1)	790	ng/µl	2.12
Vertix10_CTRL(24hpi2)	861.4	ng/µl	2.12
Vertix10_CTRL(24hpi3)	655	ng/µl	2.15
Vertix10_CTRL(48hpi1)	732.3	ng/µl	2.15

Vertix10_CTRL(48hpi2)	1038.7	ng/µl	2.11
Vertix10_CTRL(48hpi3)	1230.1	ng/µl	2.15
Vertix10_CTRL(120hpi1)	969.4	ng/µl	2.13
Vertix10_CTRL(120hpi2)	843.4	ng/µl	2.13
Vertix10_CTRL(120hpi3)	932.4	ng/µl	2.12
Vertix10_INF(12hpi1)	833.6	ng/µl	2.12
Vertix10_INF(12hpi2)	944.6	ng/µl	2.15
Vertix10_INF(12hpi3)	697.1	ng/µl	2.11
Vertix10_INF(24hpi1)	1421.2	ng/µl	2.12
Vertix10_INF(24hpi2)	1284.1	ng/µl	2.09
Vertix10_INF(24hpi3)	1271.4	ng/µl	2.14
Vertix10_INF(48hpi1)	1652.6	ng/µl	2.12
Vertix10_INF(48hpi2)	1805.9	ng/µl	2.11
Vertix10_INF(48hpi3)	1510.5	ng/µl	2.09
Vertix10_INF(120hpi1)	1947.9	ng/µl	2.10
Vertix10_INF(120hpi2)	1635.2	ng/µl	2.12
Vertix10_INF(120hpi3)	1733	ng/µl	2.12
